# Comparative Analysis of Physicochemical Properties and Microbial Composition in High-Temperature *Daqu* With Different Colors

**DOI:** 10.3389/fmicb.2020.588117

**Published:** 2020-11-27

**Authors:** Ling Deng, Xiang Mao, Dan Liu, Xin-Qiang Ning, Yi Shen, Bo Chen, Hong-Fang Nie, Dan Huang, Hui-Bo Luo

**Affiliations:** ^1^College of Bioengineering, Sichuan University of Science and Engineering, Yibin, China; ^2^Key Laboratory of Liquor Making Biotechnology and Application of Sichuan Province, Yibin, China; ^3^Sichuan Langjiu Co., Ltd., Gulin, China

**Keywords:** high-temperature *Daqu*, microbial communities, physicochemical and enzymatic properties, functional prediction, different colors

## Abstract

High-temperature *Daqu*, also called Jiang-flavor *Daqu*, is the saccharifying and fermenting agent for brewing Jiang-flavor Baijiu. During the spontaneous solid-state fermentation of high-temperature *Daqu*, variations in temperature and moisture lead to microbial diversity and various metabolites, contributing to the different colors of high-temperature *Daqu* (customarily referred to as white *Daqu*, black *Daqu*, yellow *Daqu*, and red *Daqu* in production). We aimed to investigate the differences in microbial communities, physicochemical indices, and potential functions among different high-temperature *Daqu* with different colors (labeled as QW, QB, QY, and QR) by amplicon sequencing. We found that *Kroppenstedtia*, *Bacillus*, and *Thermoascus* were predominant in all samples; *Saccharopolyspora* and *Thermomyces* were predominant in QB and QR; and *Unclassfied_O_Eurotiales* were predominant in QY. The results on the physicochemical characteristics indicated that compared with other *Daqu* samples, QW exhibited higher protease activity and lower acidity, whereas QB showed the opposite results. QR had the highest esterification yield, and QY exhibited the highest saccharification but lowest esterification yield. Functional prediction demonstrated that the higher abundances of genes encoding bacterial enzymes of QW and QY were related to the considerably higher abundances of *Kroppenstedtia* in QW (59%) and QY (87%), respectively. The highest abundance of *Thermomyces* in QB (80%) contributed to the highest abundance of genes encoding fungal enzymes in QB. This study revealed the microbial and functional dissimilarities of color-based high-temperature starters and helped facilitate the liquor fermentation process.

## Introduction

Baijiu (Chinese liquor), one of the six world-famous distilled spirits, is the most widely consumed beverage (over 13 billion liters in 2016). It is produced using a special brewing process and exhibits a distinctive flavor (Liu and Sun, 2018). Baijiu may be classified based on its special flavor characteristics into three major types: Jiang-flavor liquor, Strong-flavor liquor, and Light-flavor liquor. Among the three categories, the Jiang-flavor liquor involves the most complex and distinct brewing process; it is also the most widely consumed liquor (Zhao et al., 2016). Its flavor is similar to that of strong soy sauce and has a lingering aftertaste. The Jiang-flavor liquor is manufactured using two key processes: high-temperature *Daqu* preparation and liquor fermentation (Gan et al., 2019). High-temperature *Daqu* provides various enzymes and microbial metabolites, which are important sources of the Jiang flavor during liquor fermentation (Wang et al., 2019).

**FIGURE 1:**
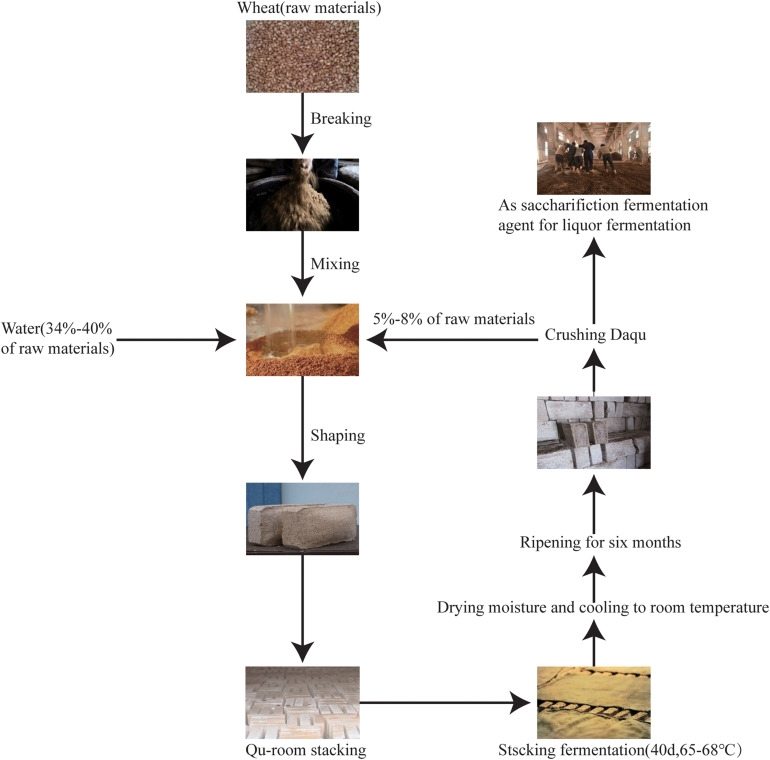
Preparation of high-temperature *Daqu* samples.

According to the maximum temperature during fermentation, *Daqu* is classified into three types: low-temperature *Daqu* (45–50°C), medium-temperature *Daqu* (50–60°C), and high-temperature *Daqu* (60–65°C) (Wang et al., 2011). High-temperature *Daqu* (Figure 1) is prepared using three principal processes: (i) shaping, (ii) fermentation (for about 1 month), and (iii) ripening (for about 6 months). During shaping, wheat grains are crushed, mixed with water and the source *Daqu* powder (high-quality *Daqu* from last year), and pressed into a brick shape. During fermentation, the starter bricks are placed in a room (the “Qu-room”) for spontaneous fermentation. The first turning (collecting and stacking scattered pieces into multiple layers) of the brick is conducted when the mold grows on the surface of the *Daqu* starter, and the second turning occurs 7–8 days after the first turning. The starters are cultured in the Qu-room for 25 days, removing the moisture, and then heated to 65–68°C for stacking and fermentation for 40 days. Once the fermentation is completed, *Daqu* is stored for 8–10 days to reduce the moisture content to 15% and to allow cooling to room temperature. During ripening, all fermented starters are stored in an open room for 6 months and then crushed and mixed to start the liquor fermentation (Wang et al., 2019). The processing of high-temperature *Daqu* is the key to brewing the Jiang-flavor liquor. High-temperature *Daqu* mainly varies from other *Daqu* starters (low-temperature and middle-temperature *Daqu*) not only in temperature but also in complexity of microbial community structure and succession, complexity of enzyme systems, and soy sauce content in *Daqu* (Jiang et al., 2018). Under this high-temperature method, environmental pressure contributes to the formation of a distinct microbial community structure and an enzyme system of high-temperature *Daqu* (Huang et al., 2017). The microbial communities and rich enzymes in *Daqu* facilitate liquor fermentation and determine the final distinct flavor.

During the spontaneous fermentation of high-temperature *Daqu*, heterogeneous environmental factors of the original locations of the Qu-room, such as temperature and moisture, contribute to the colors of *Daqu*: white, yellow, red, and black (Ban et al., 2015). Yellow *Daqu* generally exhibits the best fermentation characteristics, followed by white *Daqu* and black *Daqu* (Qin et al., 2015). Suitable temperatures at the pre-fermentation stage and sufficient drying at the late fermentation stage contribute to the production of yellow *Daqu*; low temperatures at the pre-fermentation stage and poor drying at the late fermentation stage yield white *Daqu*; rapidly rising temperatures at the early fermentation stage and poor drying at the late fermentation stage result in black *Daqu*, indicating the worst quality. Red *Daqu*, with red color in the inner of *Daqu*, mostly comes from black *Daqu* in the actual production process. At appropriate high temperatures and humidity levels, a mild Maillard reaction occurs, producing yellow *Daqu* with a strong soy sauce flavor (Gan et al., 2019). During a Maillard reaction, an amino acid and a reducing sugar are heated to produce brown compounds, which give *Daqu* its color (Zheng et al., 2011). Free amino acids are found mostly in black *Daqu*, and in decreasing amounts, in yellow and white *Daqu* (Zhang et al., 2019). Different stages of *Daqu* fermentation vary in temperature, which potentially contributes to the formation of different microbial communities and enzyme systems. Different microorganisms and enzyme systems produce different metabolites which gives the flavors of substances under different temperatures (Cui, 2007). Zhang L. et al. (2014) identified temperature and moisture as factors influencing the growth of microorganisms and the enrichment of different microbial species during fermentation. Despite the many studies conducted, the differences in microbial communities, physicochemical and enzymatic properties, and potential functions among *Daqu* with different colors remain inconclusive.

Various methods have thus far been used to analyze microbial structures and functions during solid-state fermentation of *Daqu*. Numerous studies on Baijiu, including Strong-flavor liquor, Light-flavor liquor, and Fen liquor, have revealed the mechanism underlying their traditional fermentation processes (Li et al., 2013; Tao et al., 2014; Zhang X. et al., 2014; Hong et al., 2016; Huang et al., 2017; Tang et al., 2019; He et al., 2020). Recent studies demonstrated that *Bacillales*, *Enterobacteriales*, and *Lactobacillales* in Jiang-flavor *Daqu*, *Weissella*, *Leuconostoc*, and *Lactobacillus* in Strong-flavor *Daqu*, and *Lactobacillus*, *Bacillus*, *Kroppenstedtia*, *Weissella*, *Pantoea* in Light-flavor *Daqu* were the dominant bacteria. *Candida*, *Trichoderma*, *Aspergillus*, and *Thermomyces* in Jiang-flavor *Daqu*, *Thermoascus*, *Candida*, *Wickerhamomyces*, and *Thermomyces* in Strong-flavor *Daqu*, and *Pichia*, *Saccharomycopsis*, and *Aspergillus* in Light-flavor *Daqu* were the dominant fungal genera (Xiao et al., 2017; Wang et al., 2018; Jin et al., 2019). Yu et al. (2020) found that the three different temperature *Daqu* had little significant difference in moisture. Acidity of high-temperature *Daqu* was significantly higher than other *Daqu* (*p* < 0.05) while saccharification and liquefaction of high-temperature were strongly lower than other *Daqu* (*p* < 0.05).

In the current study, we used amplicon sequencing to analyze the microbial diversity of different colorful high-temperature *Daqu* and predicted the functional profiles based on KEGG pathway analysis by using the software Phylogenetic Investigation of Communities by Reconstruction of Unobserved States (PICRUSt2) (Langille et al., 2013). We aimed to identify the microbial structures and thereby distinguish four high-temperature *Daqu* starters with different colors. We also intended to distinguish microbes among four samples by their variations in physicochemical characteristics and enzymatic properties. By adjusting the optimal mixing ratio of different *Daqu* starters, the quality of Jiang-flavor liquor was improved.

## Materials and Methods

### Sampling

We collected four types of high-temperature *Daqu* starters with different colors from a Jiang-flavored Baijiu enterprise in Luzhou, Sichuan, China (105°81′34.7″ E, 28°03′8.67″ N). On the basis of the color in appearance of *Daqu*, these types were usually labeled as white *Daqu* (QW), black *Daqu* (QB), yellow *Daqu* (QY) in production. Red *Daqu* (QR), mostly coming from black *Daqu*, was labeled according to the internal color of *Daqu*. Supplementary Figure 1 showed four *Daqu* with different colors. We randomly collected 12 starters (three *Daqu* bricks of per color) after fermentation finishing. Three whole *Daqu* starters of each color were then powdered, mixed, and pooled into sterile bags as an experimental *Daqu* powder sample (500 g). We collected and stored four samples at 4°C for the analysis of physicochemical and enzymatic properties (400 g) and −20°C for DNA extraction (100 g).

### Analysis of Physicochemical and Enzymatic Properties

We evaluated the four *Daqu* samples with respect to eight physicochemical and enzymatic properties: acidity, moisture, amino acid nitrogen, saccharification, liquefaction, protease activity, fermenting ability, and esterifying ability. The moisture of Daqu was determined using dry/wet weight measurement at 105°C. The total titratable acidity was determined by titration with 0.1 M NaOH exhibiting a titration endpoint of pH 8.2. The measurement of saccharification, liquefaction, protease activity, esterification, and fermenting power was determined as previously described (Shen, 2007). The detailed methodology is described in the Supplementary Material. Saccharification was defined as 1mg glucose hydrolyzed from soluble starch for a hour by 1.0 g starter at 40°C pH4.6. Liquefaction was defined as the mass (g) of soluble starch liquefied for a hour by 1.0 g starter at 60°C pH6. Protease activity was defined as the mass (ug) of tyrosine hydrolyzed from casein per minute by 1.0 g starter at 40°C pH 3.0. Esterification was defined as the mass (mg) of ethyl caproate produced by 1 g starter at 30–32°C for 100 h. Fermenting power was defined as the mass (g) of CO_2_ produced from fermentable sugars for 72 h by 0.5 g starter at 30°C. The amino acid nitrogen content was determined according to the general analytical method QB/T 4257–2011 Methods (People’s Republic of China Professional Standard, 2011).

### DNA Extraction and PCR Amplification

Genomic DNA was extracted from the microbial communities of the four *Daqu* samples by using the E.Z.N.A.^®^ Soil DNA Kit (Omega Bio-Tek, Norcross, GA, United States) as instructed by the manufacturer. The DNA extract was checked for quality on 1% agarose gel, and the DNA concentration and purity were determined using the NanoDrop 2000 UV-vis spectrophotometer (Thermo Scientific, Wilmington, United States). The V4 regions of the bacterial 16S rRNA gene and the internal transcribed spacer (ITS1) regions of fungal rRNA genes were amplified using the primers 338f/806r (5′-ACTCCTACGGGAGGCAGCAG-3′/5′-GGACTACHVGGGTWTCTAAT-3′) and ITS1f/2043R (5′-C TTGGTCATTTAGAGGAAGTAA-3′/5′-GCTGCGTTCTTCATC GATGC-3′) with barcodes, respectively. PCR amplification of the gene was performed: the initial denaturation was conducted at 95°C for 3 min, followed by 27 cycles of denaturing at 95°C for 30 s; annealing at 55°C for 30 s and extension at 72°C or 45 s; single extension at 72°C for 10 min; and ultimately at 4°C. The PCR mixtures contained the following: 4 μL of 5 × TransStart FastPfu buffer, 2 μL of 2.5 mM dNTPs, 0.8 μL of the forward primer (5 μM), 0.8 μL of the reverse primer (5 μM), 0.4 μL of TransStart FastPfu DNA Polymerase, DNA 10 ng of the template, and up to 20 μL ddH_2_O.

### Illumina MiSeq Sequencing

The polymerase chain reaction (PCR) product extracted from 2% agarose gel was purified using the AxyPrep DNA Gel Extraction Kit (Axygen Biosciences, Union City, CA, United States) in accordance with the instructions provided by the manufacturer and determined using QuantiFluor^TM^-ST (Promega, United States). The library was ultimately sequenced on the Illumina MiSeq2500 platform.

### Processing of Sequencing Raw Data

Raw gene sequencing reads were demultiplexed, quality-filtered using Trimmomatic, and merged by FLASH under the following criteria: (i) 300 bp reads were truncated at any site receiving an average quality score of <20 over a 50 bp sliding window, and truncated reads shorter than 50 bp were discarded; (ii) barcodes exactly matched, 2 nucleotides were mismatched during primer matching, and reads containing ambiguous characters were removed; and (iii) only overlapping sequences longer than 10 bp were assembled in accordance with their overlapped sequence. Reads that could not be assembled were discarded. Operational taxonomic units (OTUs) with 97% similarity cutoff were clustered using UPARSE (version 7.1) (Edgar, 2013), and chimeric sequences were identified and removed using the UCHIME algorithm. Taxonomic assignment was conducted using RDP Classifier^1^ (Wang et al., 2007). Alpha diversity indices Chao1, Shannon, Goods coverage were performed using the software package mothur to reflect the diversity and richness of microbial communities in different samples (Schloss et al., 2011).

### Statistical Analysis

The statistical significance (*P* ≤ 0.05, Duncan’s test) of differences among the original samples was determined using ANOVA in SPSS 25.0. Fisher’s exact test was used to generate a bar plot displaying differences between two sets of samples. PICRUSt2 was used to predict microbial functions based on the KEGG pathway database^2^. Redundancy analysis (RDA) was performed to reveal the correlations between microbiota at the genus level and physicochemical and enzymatic properties by using the Canoco 4.5 software. RDA, mainly used to reflect the relationship between microbes and environmental factors, is a principal component analysis (PCA) with environmental factors constrained. The sample and environmental factors can be reflected on the same two-bit sequence diagram. The cos showed a positive (cos > 0) or negative (cos < 0) relationship among the samples, and the projector distance of variables in the direction of the genera represents their quantitative relation (Ramette, 2007; Lee et al., 2015).

## Results

### Microbial Diversity Analysis

We found 107272 valid sequences from bacteria (with an average of 26818 bacterial sequences per sample) and 122172 valid sequences from fungi (with an average of 30543 fungal sequences per sample) in five Jiang-flavor *Daqu* samples. We determined the alpha and beta diversities of the bacterial and fungal communities with normalized sequences. Table 1 lists the α-diversity indices of the microbial communities. The results suggested that the species richness (Chao1) and diversity (Shannon and Simpson) of bacteria were higher than those of fungi in the majority of the samples. QR showed the highest bacterial values but the lowest fungal values, and QY had the highest fungal values. QB exhibited higher microbial species richness and diversity, compared with QW and QY.

**TABLE 1:** α-diversity indices for microbial communities among samples.

Samples	Abundance indices				Diversity indices			
		
	Chao1		ACE		Shannon		Simpson	
				
	Bacteria	Fungi	Bacteria	Fungi	Bacteria	Fungi	Bacteria	Fungi
QW	49.50	21.50	55.19	22.12	1.57	0.99	0.36	0.48
QB	63.00	18.00	63.80	28.00	2.14	0.58	0.16	0.67
QY	51.17	29.00	52.94	32.58	1.11	1.10	0.40	0.38
QR	77.50	18.00	77.15	20.16	2.04	0.61	0.21	0.71

**FIGURE 2:**
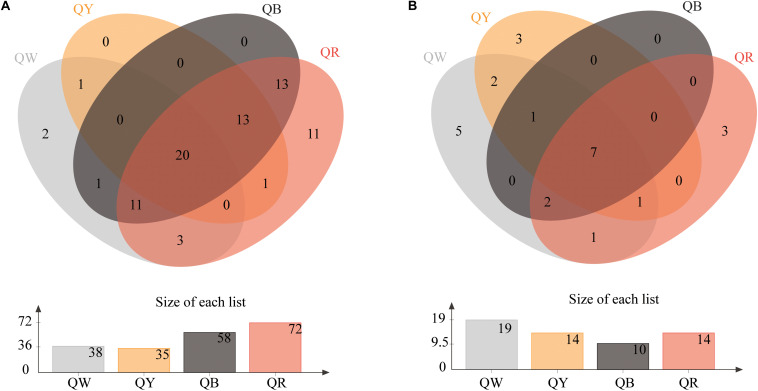
Venn diagram of OTUs in four samples. **(A)** Shows the results for bacteria, and **(B)** presents the results for fungi.

The Venn diagrams of Figure 2 show the microbial composition at the OTU level in four different samples. A total of 75 bacterial OTUs were found in the four *Daqu* samples, with QR having the largest number of OTUs (72), QW had 35 OTUs, and QY had 35 OTUs; the number of OTUs in each of QW and QY was significantly lower than that in QR. QB and QR shared about 78% OTUs (57), which comprised a large proportion. Meanwhile, 25 fungal OTUs were found in the four *Daqu* samples. The four samples shared 7 OTUs, which comprised a small percentage, in all samples. The largest number of OTUs was found in QW (19), followed by QY (14), QR (14), and QB (10).

### Analysis of Microbial Composition and Difference

**FIGURE 3:**
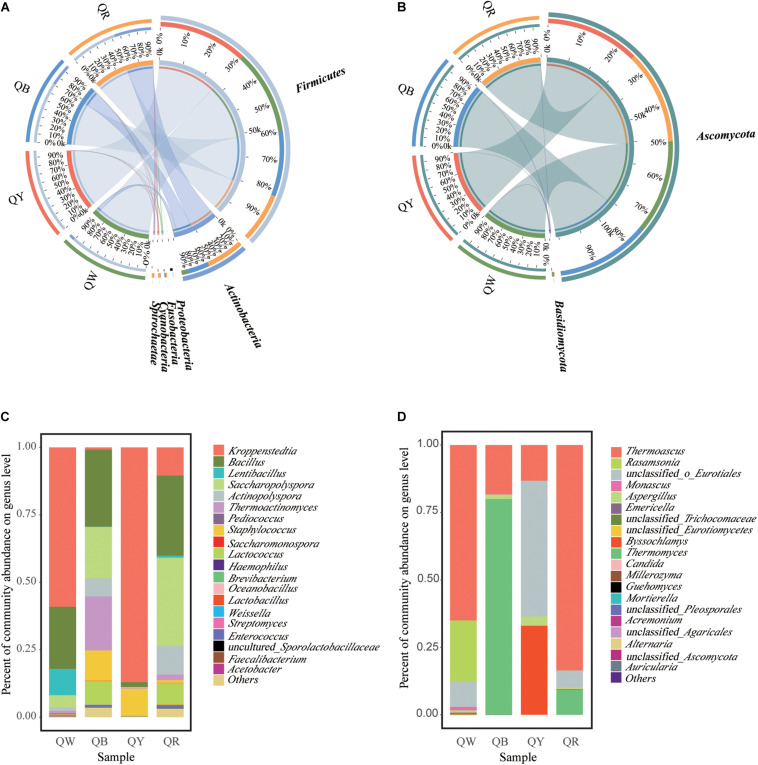
Microbiota composition. Graphs for bacteria **(A)** and fungi **(B)**, generated using the software CIRCUS. The left semicircle represents the phylum composition of each group. The right semicircle shows the distribution of each phylum in the disparate groups. Bar plots of bacterial **(C)** and fungal **(D)** communities at the genus level. “Others” includes all taxa less than 1%.

The differences in microbial communities at the phylum level among the four *Daqu* samples became highly evident by using Circos (Ye et al., 2014). Firmicutes and Actinobacteria were the main bacterial phyla (Figure 3A), and this finding was consistent with a previous study (Gan et al., 2019). Ascomycota was the predominant fungal phylum, comprising 97.2% of the total fungal population (Figure 3B). Meanwhile, the dissimilarities in genera in the four samples were apparent (Figures 3C,D). *Kroppenstedtia, Bacillus*, and *Saccharopolyspora* were the most predominant bacterial genera in the four samples. The dissimilarities among the four samples were as follows: *Kroppenstedtia* was in QW (59%), QY (87%), QB (0.8%), and QR (10%). *Bacillus* was in QW (23%), QY (1.8%), QB (29%), and QR (30%). *Saccharopolyspora* was in QW (4.5%), QY (0.3%), QB (19%) and QR (33%). *Thermoactinomyces* was in QW (1%), QY (0.2%), QB (20%) and QR (2%). Meanwhile, *Thermoascus*, *Thermomyces*, and *unclassfied_o_Eurotiales* were the major fungal genera in the four samples; *Thermoascus* was in QW (65%), QY (13%), QB (18%) and QR (84%). *Thermomyces* was in QW (0%), QY (0%), QB (80%), and QR (9%). *Unclassfied_o_Eurotiales* was in QW (9%), QY (50%), QB (0%) and QR (6%). *Byssochlamys* was in QW (0%), QY (33%), QB (0%) and QR (0.3%).

**FIGURE 4:**
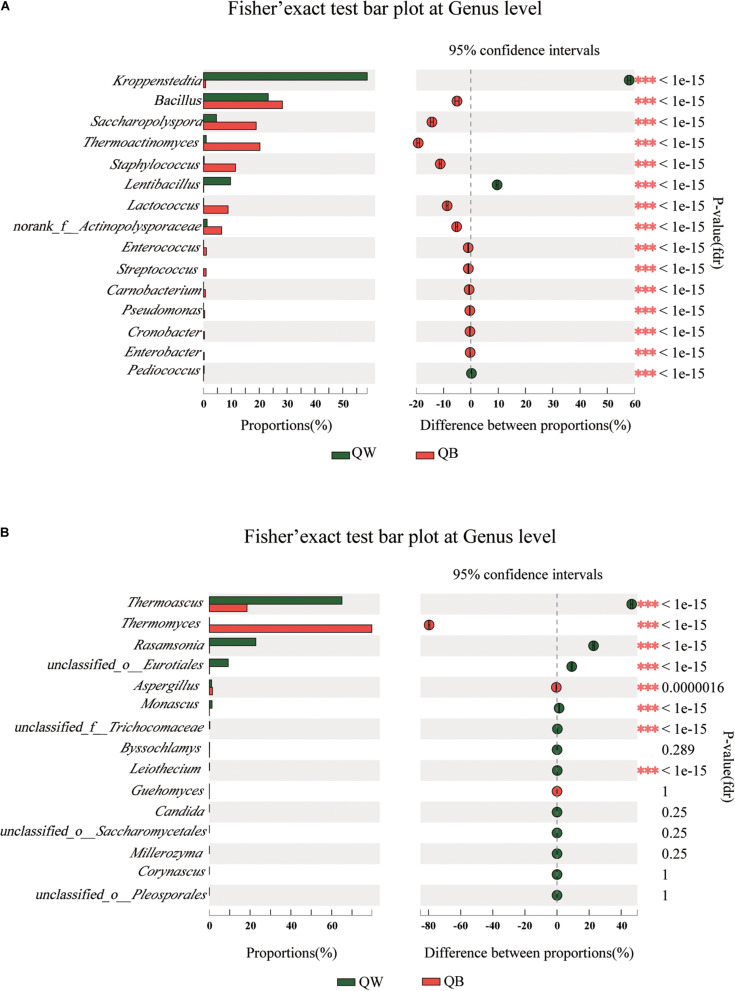
Bar plots generated using Fisher’s exact test results for the four samples. The 15 most abundant genera (**A**, bacteria; **B**, fungi) in QW and QB are depicted. The *y*-axis represents the species name at a certain classification level, each column corresponding to a species represents the relative abundance of the species in each sample, and different colors represent different samples; the middle region is the set confidence interval, the value corresponding to the dot represents the difference of the relative abundance of species in the two samples, the dot color shows the color of the sample with larger species richness, and the type I area on the dot Are the upper and lower limits of the difference. *Correlation is significant at the 0.05 level. **Correlation is significant at the 0.01 level. ***Correlation is significant at the 0.001 level.

We conducted a *post hoc* analysis with Fisher’s exact test (two sides) on the four samples. Fisher’s exact test was used to compare the species richness difference between the two samples, and the significance of species difference in the two contrast samples could be obtained through this analysis(Koul et al., 2012; Moukharskaya et al., 2016). The plot presents a pairwise comparison of microbial communities between two sets of samples. We obtained the results for microbiological comparisons between two of the four samples: two of the twelve figures are shown as examples (Figures 4A,B) and the other ten results are shown in Supplementary Figures 2, 3. Significant differences (*p* < 0.001) among the four samples were determined. Bacterial or fungal genera, showed a significant difference in abundance with other genera in two of the three comparisons, were defined as the different species when one *Daqu* sample was compared with the other three samples. With regard to bacterial genera, significant differences were found in the following species: *Lentibacillus* in QW; *Thermoactinomyces*, *Staphylococcus*, and *Lactococcus* in QB*; Kroppenstedtia* in QY; and *Saccharopolyspora* in QR. With regard to fungal genera, the following were considered as distinct species: *Rasamsonia* and *Monascus* in QW; *Thermomyces* in QB; *unclassfied_o_Eurotiales*, *Byssochlamys*, and *Aspergillus* in QY; and *Thermoascus* in QR.

### Physicochemical and Enzymatic Characteristics

Physical and chemical properties are the reference for identifying the quality of *Daqu*, and microbial function in the starters is related to physical and chemical properties (Gan et al., 2019). Differences in the eight physicochemical characteristics (Duncan-test *P* < 0.01) among the original samples were observed (Table 2). QW exhibited the highest protease activity (168.06 u/g) and the lowest acidity (13.27 mmol/10 g), whereas QB had the lowest protease activity (33.02 u/g) and the highest acidity (16.93 mmol/10 g). Except for its esterifying ability (35.54 mg/g) and amino acid nitrogen (1.39 g), QY reflected moderate physical and chemical properties. QR showed the highest esterifying ability (143.87 mg/g) but the lowest fermenting ability (0.15 g/0.5g.72 h). These results indirectly reflected microbial abundance and metabolism in *Daqu*.

**TABLE 2:** Eight physical and chemical properties evaluated in the four high-temperature Daqu samples.

Properties	QW	QB	QY	QR
Moisture (%)	12.86 ± 0.03A	10.58 ± 0.11B	12.54 ± 0.02A	13.00 ± 0.10A
Acidity (mmol/10 g)	13.27 ± 0.00D	16.93 ± 0.26A	15.56 ± 0.00B	14.64 ± 0.00C
Amino acid nitrogen (g/Kg)	2.03 ± 0.01A	1.48 ± 0.06C	1.39 ± 0.02C	1.53 ± 0.01B
Liquefaction (g/g.h)	137.43 ± 0.05A	133.17 ± 0.05C	137.12 ± 0.07B	137.09 ± 0.16B
Saccharification (ma/g.h)	16.40 ± 0.26C	44.00 ± 0.36B	56.00 ± 0.09A	58.00 ± 1.25A
Protease activity (u/g)	168.06 ± 0.02A	33.02 ± 0.00D	67.28 ± 0.01B	41.61 ± 0.03C
Fermenting ability(g/0.5g.72h)	0.26 ± 0.01C	0.32 ± 0.01A	0.29 ± 0.01B	0.15 ± 0.01D
Esterifying ability (mg/g)	42.01 ± 0.69C	84.76 ± 0.48B	35.54 ± 0.60D	143.87 ± 0.33A

*ABCD is a significant marker and A indicates the maximum. The same mark letter indicates no significant difference, and different letter marks indicate significant difference.*

### Correlations Between Predominant Communities and Physicochemical Properties of *Daqu* Samples

**FIGURE 5:**
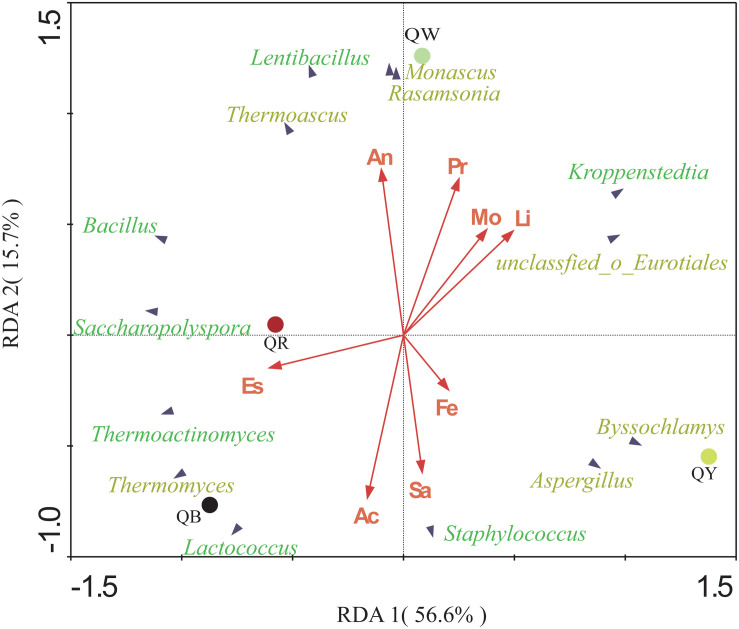
Redundancy analysis (RDA) of the different genera and the eight physical and chemical properties, including acidity (Ac), moisture (Mo), amino acid nitrogen (An), saccharification (Sa), liquefaction (Li), protease activity (Pr), fermenting ability (Fe), and esterifying ability (Es). Bacterial genera are in green, and fungal genera are in yellow.

The potential correlations between the predominant genera with significant differences (results of the above Fisher’ exact test, *p* < 0.001) and eight indices in four *Daqu* samples were plotted using RDA analysis (Figure 5). These indices included moisture, acidity, amino acid nitrogen, liquefaction, saccharification, protease activity, fermenting ability, and esterifying ability. From the diagram, the relationship between the sample and microbe distribution and environmental factors can be seen intuitively (Ramette, 2007). Moisture and liquefaction showed positive correlations with *Kroppenstedtia*. Saccharification exhibited a strong positive correlation with *Staphylococcus.* Meanwhile, Esterification was positively correlated with *Thermoactinomyces* near QR, which was consistent with the highest esterification (143.87 ± 0.33 mg/g) in QR (Table 2). Fermenting power also showed a strong positive correlation with *Byssochlamys* and *Aspergillus* near QY. Amino acid nitrogen and protease activity were positively correlated with *Rasamsonia* and *Monascus* near QW.

### Prediction of Functional Profiles for the Four *Daqu* Samples

**FIGURE 6:**
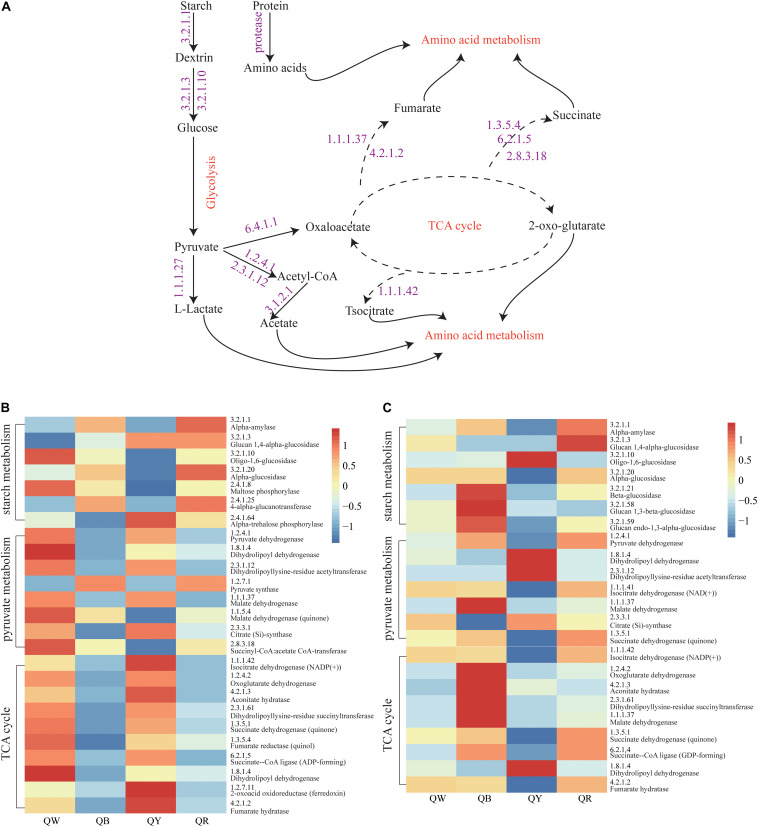
Analysis of predicted functional profiles for the four *Daqu* samples, based on the KEGG pathway. **(A)** A schematic of the metabolism of main microbes during *Daqu* brewing. **(B)** Heatmap of the high abundance of genes annotated to enzymes in starch and sucrose metabolism, pyruvate metabolism, and the tricarboxylic acid cycle (left heatmap, bacteria: right heatmap, fungi).

The abundances of genes encoding enzymes related to starch and sucrose metabolism, pyruvate metabolism, and TCA cycle, which reflected the strength of biochemical reactions during fermentation, were evaluated using the PICRUSt2 approach (Figure 6A). For the bacteria, the abundances of the encoding genes of these enzymes were different among the QB, QR, QW, and QY groups (Figure 6B). Enzymes related to starch metabolism showed more activity in QB and QR than in QW and QY; Meanwhile, pyruvate metabolism and the TCA cycle in QB and QR exhibited the opposite activities, except for pyruvate synthase (EC:1.2.7.1). For fungi, the genes encoding the enzymes related to three metabolisms (starch and sucrose metabolism, pyruvate metabolism and TCA cycle) in QB exhibited the highest abundances (Figure 6C), followed by those in QR, QW, and QY.

These findings suggested that bacteria contributed to the rich metabolic activities of QW and QY. Moreover, fungi mainly contributed to QB and QR. Figure 6B shows the general metabolic processes of the three metabolisms and related enzymes. Lactate, acetate, fumarate, succinate, and isocitrate as metabolic intermediates participate in further metabolic activities to form flavor compounds such as alcohols, aldehydes, ketones, and esters.

## Discussion

The present study focused on the differences among four high-temperature *Daqu* samples with different colors. These results can provide important information on liquor fermentation and fill the knowledge gap regarding the intrinsic dissimilarities of Jiang-flavor *Daqu* based on the color classification method.

The Chao and Shannon indices of bacterial diversity in QR were the highest, whereas those in QY were the lowest (Table 1). These results were consistent with the numbers of OTUs in QR and QY (Figure 2). *Kroppenstedtia*, *Bacillus*, and *Saccharopolyspora* were the predominant bacterial genera in the four *Daqu* samples (Figure 3C). *Thermoascus*, *Thermomyces*, *unclassfied_o_Eurotiales*, and *Byssochlamys* were the predominant fungal genera (Figure 3D). Meanwhile, the community structure of QB and QR was more similar at genus level, which further showed red *Daqu* mainly came from black *Daqu*. Dominant genera (*Kroppenstedtia*, *Bacillus*, *Thermoascus*, and *Thermomyces*) were consistent with previous studies (Jiang et al., 2018; Jin et al., 2019; Shen et al., 2020). *Byssochlamys* was rarely detected in the process of *Daqu* making. In this study, it was the dominant genus of yellow *Daqu*.

*Kroppenstedtia*, *unclassfied_o_Eurotiales*, and *Byssochlamys* were the predominant genera in QY. Fisher’s exact test results suggested that *Kroppenstedtia*, *unclassfied_o_Eurotiales*, and *Byssochlamys* were biomarkers of QY. *Kroppenstedtia* is from the family of *Thermoactinomyces*, which thrives on the high temperature and high humidity in *Daqu*. Proper high temperature and humidity levels, contributing to moderate Maillard reaction in the starters, are key in the production of QY (Gan et al., 2019). High humidity provides a foundation for high temperature and the maximum temperature of high-temperature *Daqu* reaches 60–65°C (Wang et al., 2011; Qin et al., 2015). Hu et al. (2016) also indicated that controlling the time of turning (collecting and stacking scattered pieces into multiple layers) will affects the temperature and moisture content of the starter and thus the color of *Daqu*. Many studies have found that *Kroppenstedtia* was predominant in different *Daqu* samples, but its fermentation characteristics have rarely been reported (Yao et al., 2012; Yang et al., 2020). In the current study, *Byssochlamys* was the predominant genus of mold, which can degrade starch or cellulose in raw materials. Wang et al. (Wang et al., 2016) indicated that *Byssochlamy*s was rarely detected in the production of *Daqu*. *Bacillus, Thermoactinomyces*, and *Thermomyces* were the main genera in QB; meanwhile, *Thermoactinomyces* occurred in small quantities in QW. Shen et al. (2019) showed that a long high-temperature stage contributed to the dominance of these thermotolerant genera in QB; they also increased the activity of thermostable enzymes and intensified maillard and caramelization reactions to form numerous black or dark brown compounds; by contrast, lower temperatures inhibited the growth of thermophilic fungi in QW (Shen et al., 2019). The predominant genera in QR were *Bacillus* and *Sacchropolyspora*, and the relative abundance of *Monascus* in QR was 0.003%. Shen (2007) described that a large *Monascus* count in the inner part of red *Daqu*. This was inconsistent with our findings. Our experiments found that the red pigment did not indicate the presence of *Monascus* but instead was produced by other bacteria. Previous studies also found *Bacillus subtilis* and *Bacillus sarcina* can produce red pigment and may play an important role in liquor fermentation and red color in *Daqu* center (Zhao et al., 2009; Wei et al., 2014). Gan et al. (2019) suggested that *Saccharopolyspora* was essential for producing flavoring substances during Jiang-flavor liquor production. These findings prompt the use of colorful *Daqu* during Jiang-flavor liquor fermentation. Further research is necessary to reveal specific mechanisms.

Moisture, acidity, liquefaction, and saccharification reflect the maturity of *Daqu*. Amino acid nitrogen, protease activity, fermenting power, and esterification reflect the quality of *Daqu* (Hu et al., 2017). The average moisture content was 12.5% which reached the limit (<13%) for the storage period (Chui et al., 2011). The difference in moisture content suggests different levels of water discharge, which is related to the spread of mycelium during *Daqu* production. QB showed the lowest moisture, suggesting that QB endured a longer high-temperature stage and showed more biochemical reactions, compared with other samples during fermentation. Acidogenic microorganisms that degrade starch, fat, and protein by organic acid metabolism via the TCA pathway mainly caused the acidity of *Daqu* (Shen et al., 2005). QW exhibited the highest amino acid nitrogen, highest protease activity, and lowest acidity of QW, contrary to those of QB. These findings were attributed to the production of amino acid and peptide fractions associated with protein degradation in the raw materials (Awasthi et al., 2014). High protease activity contributed to more protein degradation, leading to the production of alkaline substances. *Staphylococcus* had a higher abundance in QB than in other types (Figure 3C). The contradictory results for QW and QB were attributable to the aforementioned findings. Two important indicators of *Daqu*, the liquefaction and saccharification, reflect the enzymes and microorganisms in *Daqu* for converting starch into sugar (Wang et al., 2015). QB and QW exhibited the lowest liquefaction and saccharification, respectively; QY exhibited the highest saccharification, close to that of QR. These findings were inconsistent with a previous report (Qin et al., 2015). The esterification ability of *Daqu* was related to the formation of ester compounds, such as ethyl hexanoate, ethyl butyrate, ethyl acetate, and ethyl lactate in Baijiu, and the fermenting ability reflected the ability of the microbes to produce alcohol (Wang et al., 2015; Fan et al., 2018). The highest esterification of QR could be associated with its bacterial diversity (Table 1). The highest fermenting efficiency of QB further suggested that increased metabolic activity leads to large quantities of carbon dioxide in QB.

The physicochemical characteristics and the microbial communities in starters affect each other (Cai et al., 2018). Different temperature contributes to different microbial community and enzymatic systems, which produce different metabolites. Different metabolites react at different temperatures to form different aroma compounds (Cui, 2007). QB showed a positive correlation with *Thermomyces*, which may be related to the longer high temperature of QB. *Thermoactinomyces* showed a significant positive correlation with esterification near QR. QR had the highest esterification but the abundance of *Thermoactinomyces* was higher in QB. This inconsistency in the result is reasonable is attributed to the complexity of interactions between microorganisms, as well as the complexity of enzymatic systems in *Daqu*. Compared with QB, QY, and QR, QW showed a stronger positive correlation with amino acid nitrogen (Table 2).

We analyzed the potential function profile of the four Jiang-flavor *Daqu* samples by using PICRUSt2, based on the KEGG pathway to explain the differences between the samples (Figure 6). Starch hydrolyzing and glucose producing, related to liquefaction and saccharification, are the essential processes in Jiang-flavor liquor fermentation. Pyruvate, the production of glucose disposal, plays a significant role in several metabolic pathways of metabolites in *Daqu*, such as lactate, acetate, and ethanol. The TCA cycle is not only the final metabolic pathway of the three nutrients (carbohydrate, lipid, and amino acid) but also the junction of carbohydrate, lipids, and amino acids metabolism. The intensity of these metabolic activities may indirectly be related to the color depth of *Daqu*. Thus, colorful *Daqu* had different contributions to the formation of the soy-sauce flavor. Figure 6 suggested that the abundances of genes encoding enzymes, related to starch metabolism, are richer in QR; this finding was consistent with the higher liquefaction and saccharification in QR (Table 2). Previous studies have shown that the amylase and protease secreted by *Bacillus* convert starch and proteins into glucose and amino acids, facilitating the formation of flavor compound precursors during liquor fermentation (Wang et al., 2011; Zheng et al., 2012). Therefore, a higher abundance of *Bacillus* in QR could increase the chance of having a higher abundance of genes encoding enzymes related to starch metabolism. For the bacteria, the abundances of genes encoding enzymes, related to pyruvate metabolism and the TCA cycle, were higher in QW and QY than in QB and QR. This finding might coincide with the considerably higher abundance of *Kroppenstedtia* in QW (59%) and QY (87%) (Figure 3C). For the fungi, the abundances of genes encoding enzymes were the richest in QB, followed by QR, QW, and QY. This finding could be associated with the abundance of *Thermomyces* in QB (80%) being the highest and that of *Thermoascus* being the highest in QR (84%), followed by QW (65%), and QY (13%) (Figure 3D). A long high-temperature stage causes the survival of only thermophilic genera such as *Thermomyces* and *Thermoascus* and the accumulation of enzymes in the early stages to intensify the Maillard reaction and caramelization, leading to the formation of more black compounds on the surface of black *Daqu* (Ban et al., 2015). Both thermophilic fungi have been reported to produce large quantities of thermophilic enzymes for carbohydrate degradation (McClendon et al., 2012). These indefinite results need to be verified using new technologies and methods such as metaOmics technologies.

## Conclusion

In conclusion, four *Daqu* samples with different colors differ in microbial composition, physicochemical and enzymatic properties, and potential metabolism function. *Kroppenstedtia*, *Bacillus*, *Thermoascus*, and *Rasamsonia* were dominant microbes in QW, and *Thermoascus* and *Rasamsonia* showed positive relationships with high protease activity and amino acid nitrogen of QW by RDA analysis. *Kroppenstedtia*, *Staphylococcus*, *Unclassfied_O_Eurotiales*, and *Byssochlamys* were predominant in QY. Meanwhile, the functional prediction results suggested that a higher abundance of genes encoding bacterial enzymes of QW and QY could be caused by a considerably higher abundance of *Kroppenstedtia* in QW (59%) and QY (87%). The community structure of QB and QR was more similar at genus level, which further indicated that QR mainly produced in QB. These findings provided new insight into the differences in color-based *Daqu*, which is essential to facilitate the liquor fermentation process and liquor quality control.

## Data Availability Statement

The datasets presented in this study can be found in online repositories. The names of the repository/repositories and accession number(s) can be found below: https://www.ncbi.nlm.nih.gov/genbank/, PRJNA662833.

## Author Contributions

H-BL, DH, YS, BC, and H-FN provided the concept, the framework, and overall support of the study. LD, XM, and DL generated the data and performed the bioinformatics analysis. LD, H-BL, DH, and X-QN wrote and edited the manuscript. All the authors contributed to the article and approved the submitted version.

## Conflict of Interest

YS, BC, and H-FN were employed by the company Sichuan Langjiu Co., Ltd. The remaining authors declare that the research was conducted in the absence of any commercial or financial relationships that could be construed as a potential conflict of interest.

## Footnotes

1 http://rdp.cme.msu.edu/

2 http://www.genome.jp/kegg/pathway.html
