# Supplementary figures and images for: Comparative Analysis of Physicochemical Properties and Microbial Composition in High-Temperature Daqu With Different Colors
Source: Front Microbiol. 2020 Nov 27;11:588117. doi: 10.3389/fmicb.2020.588117 (PMC7732550; doi:10.3389/fmicb.2020.588117)

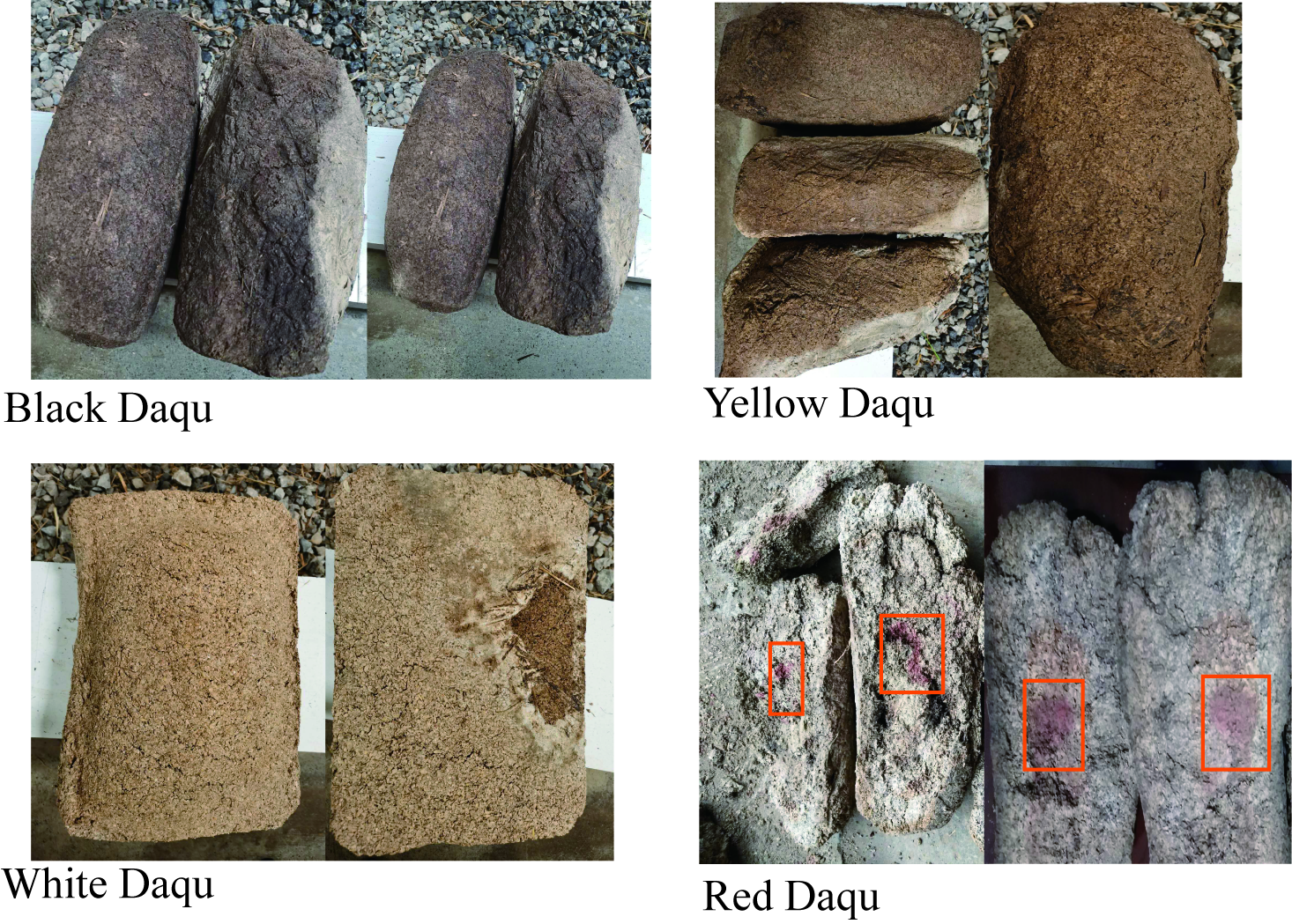

Supplement: Supplementary file 1 [file Image_1.TIF]

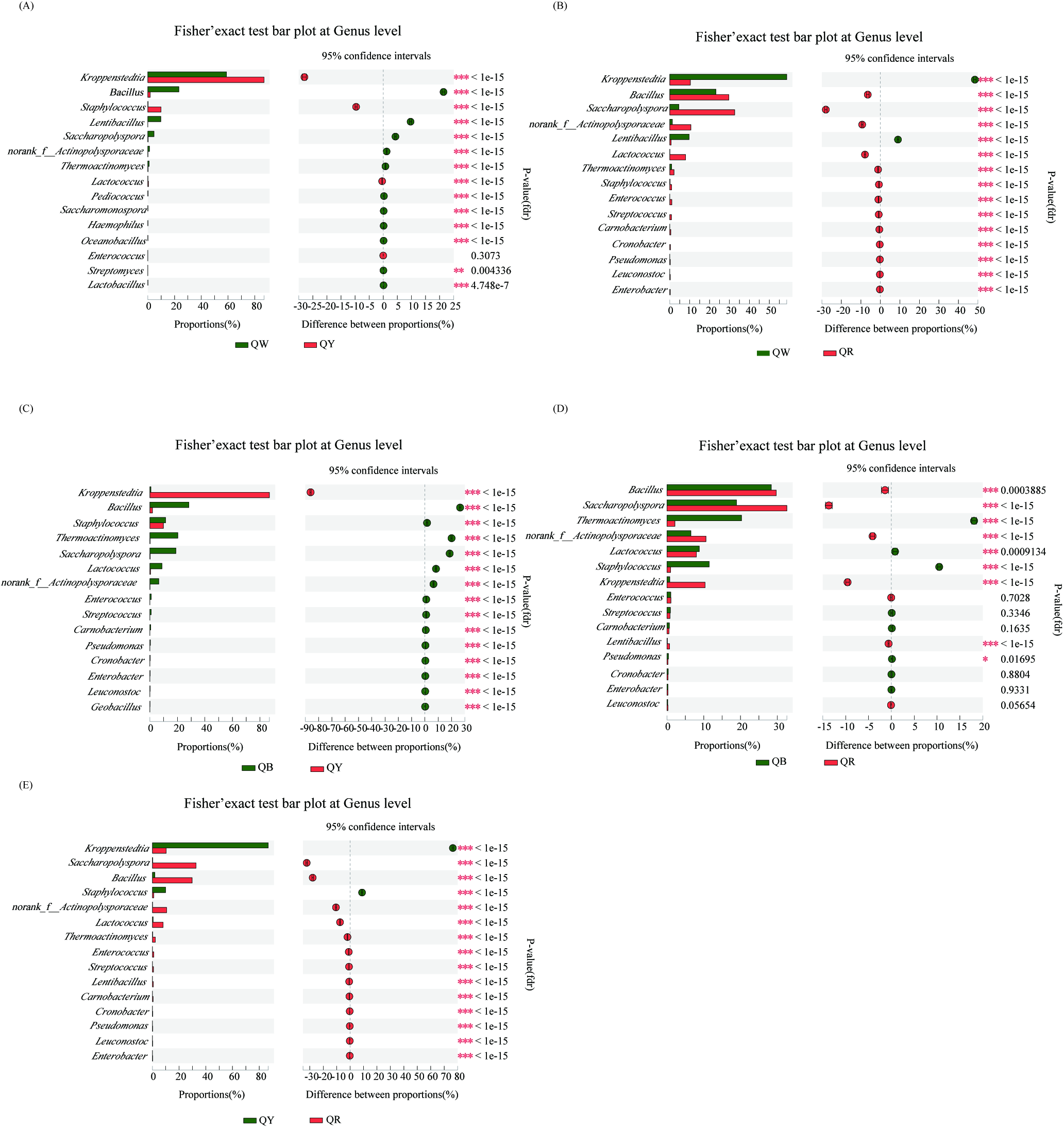

Supplement: Supplementary file 2 [file Image_2.TIF]

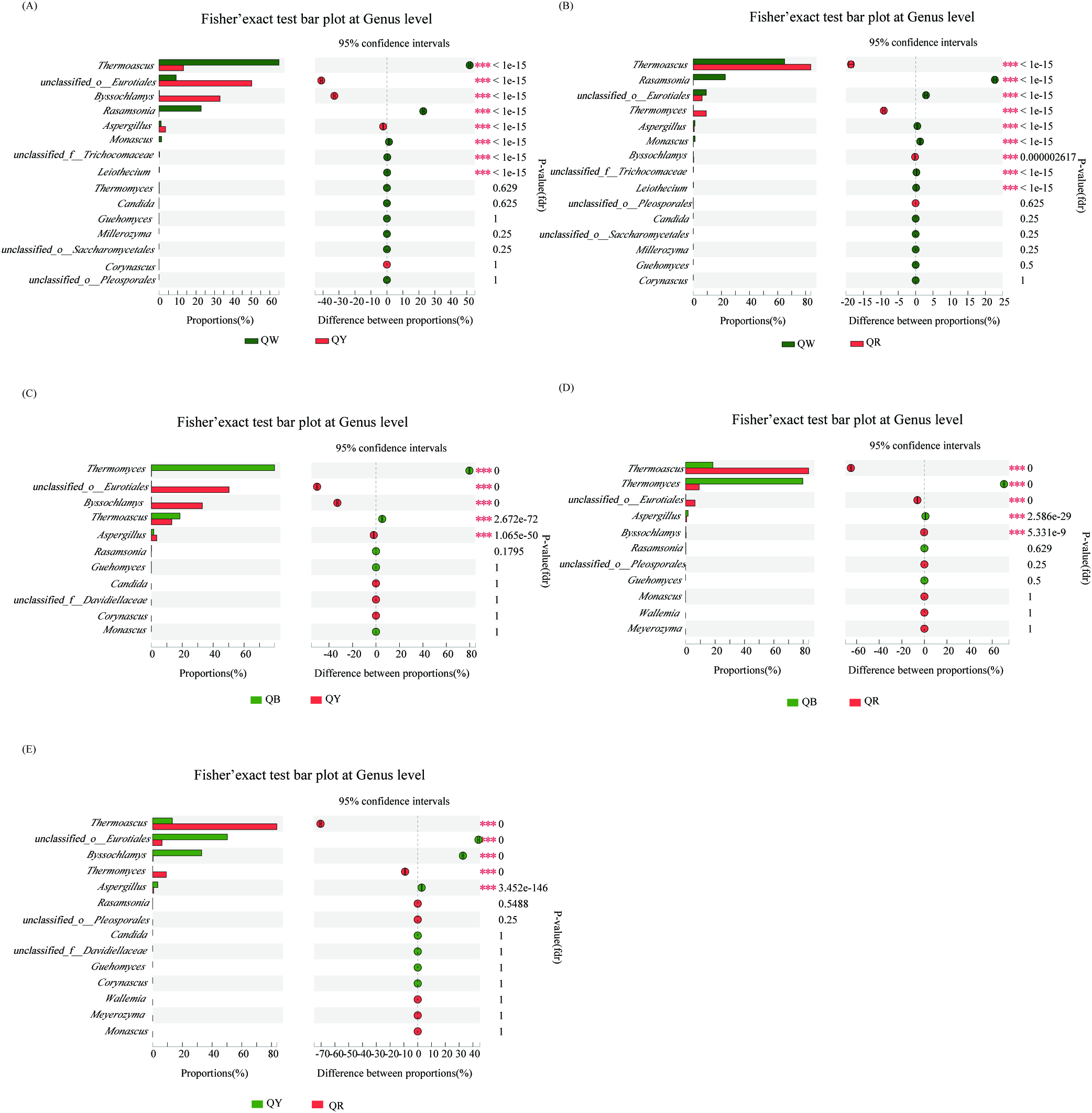

Supplement: Supplementary file 3 [file Image_3.TIF]
