# Supplementary material for: Comparative Analysis of Physicochemical Properties and Microbial Composition in High-Temperature Daqu With Different Colors
Source: Front Microbiol. 2020 Nov 27;11:588117. doi: 10.3389/fmicb.2020.588117 (PMC7732550; doi:10.3389/fmicb.2020.588117)
Supplement: Supplementary file 4 [file Data_Sheet_1.docx]

**Supplementary Material**

1. Measure methods of five enzymatic properties
2. Saccharification: 5 g starter was mixed with 85 mL water and 10 mL acetic acid - sodium acetate buffer solution, and kept in a 35°C thermostat water bath for 1h and filtered. The 10 mL filtrate was then mixed with 50 mL 2% soluble starch solution, and again kept in a 35°C thermostat water bath for 1h. The resulting solution was mixed with Fehling's solution, and titrated with 0.2% standard glucose solution until the blue color disappear. The saccharifying power was calculated with respect to the used volume of the glucose solution;
3. Liquefaction: weight 10 g starter and mix with 40°C water, add 200 mL pH 6.0 citric acid - sodium hydrogen phosphate buffer, soak for 1h (mix every 15min), filter and get the enzyme solution, mix 20 mL 2% soluble starch solution and 5 mL pH 6.0 citric acid - sodium hydrogen phosphate buffer and heat 10min in a 60°C water bath, add accurate 5 mL enzyme solution and begin timing, totally mix and take about 0.5 mL reaction mixture to a blank hole with 1.5 mL iodine solution on a white porcelain plate, stop timing when the solution color is from blue to red and finally the same as standard colorimetric solution color, calculate the liquefying power with the consumed time;
4. Protease activity: 10 g starter was mixed with 200 mL lactic acid-sodium lactate buffer solution, and kept in a 40°C thermostat water bath for 0.5 h and filtered. The 1mL filtrate was injected into a 10mL centrifuge tube (in triplicate) in a 40°C water bath for 5 minutes to preheat. 1mL of 2% casein solution was added into tube for 10 minutes and then 2mL of 0.4mol/L trichloroacetic acid was added after incubation to stop reaction. Centrifuge after 15 minutes, drawing 1mL of the supernatant into a 20mL test tube, adding 5.0mL 0.4mol/L sodium carbonate solution and 1mL Folin reagent, shaking well, and developing color in a 40℃ water bath for 20min. Measuring the optical density at a wavelength of 680nm and taking the average of the three results.
5. Esterification: 5g starter was mixed with 100ml1% hexanoic acid ethanol solution and kept at 30-32 ℃ for 100h to react. Esterified solution was mixed with 50mL water in a 250ml distillation flask for distillation. 50ml distilled solution was titrated with 0.1mol/l sodium hydroxide solution to the end point of phenolphthalein. Then add 25ml 0.1mol/l sodium hydroxide solution, refluxing and saponification in boiling water bath for 30min. After cooling, titrate with 0.05mol/l sulfuric acid solution until phenolphthalein pink disappears, calculate the esterifying power with the consumed sodium hydroxide solution.
6. Fermenting power: under aseptic condition, 1g starter and 150ml sterilized saccharified liquid were put into 250ml fermentation bottle, 10ml 5mol / L sulfuric acid solution was added into fermentation suppository, and then weighed. The fermentation bottle was placed in a 25 ℃ incubator for 48h. The fermentation bottle was taken out and shaken gently to make carbon dioxide escape, and then weighed again on the same balance, calculate the fermenting power with weight.
7. Supplementary Figures

Figure S1 Schematic diagram of high-temperature *Daqu* with different colors.

Figure S2 Bar plots generated using Fisher’s exact test results for the four samples. The bar plots present the 15 most abundant bacterial genera. (A) QW and QY; (B) QW and QR; (C) QB and QY; (D) QB and QR; (E) QY and QR.

Figure S3 Bar plots generated using Fisher’s exact test results for the four samples. The bar plots present the 15 fungal genera with the highest abundance. (A) QW and QY; (B) QW and QR; (C), QB and QY; (D) QB and QR; (E) QY and QR.
